# Functional MRI Techniques Suggesting that the Stress System Interacts with Three Large Scale Core Brain Networks to Help Coordinate the Adaptive Response: A Systematic Review

**DOI:** 10.2174/1570159X21666230801151718

**Published:** 2023-08-03

**Authors:** George Paltoglou, Charikleia Stefanaki, George P. Chrousos

**Affiliations:** 1 University Research Institute of Maternal and Child Health and Precision Medicine, Medical School, National and Kapodistrian University of Athens, “Aghia Sophia” Children's Hospital, Athens 11527, Greece;; 2 Second Department of Pediatrics, School of Medicine, National and Kapodistrian University of Athens, “A. & P. Kyriakou” Children's Hospital, Athens 11527, Greece;; 3 UNESCO Chair on Adolescent Health Care, Medical School, National and Kapodistrian University of Athens, Athens 11527, Greece

**Keywords:** Stress system, functional MRI, functional connectivity, default mode network, salience network, central executive network, mindfulness

## Abstract

**Objective:**

Synthesis of functional MRI (fMRI) and functional connectivity (FC) analysis data on human stress system (SS) function, as it relates to the dynamic function of the Salience (SN), Default Mode (DMN) and Central Executive (CEN) networks.

**Methods:**

Systematic search of Medline, Scopus, Clinical Trials.gov, and Google Scholar databases of studies published prior to September 2022 resulted in 28 full-text articles included for qualitative synthesis.

**Results:**

Acute stress changes the states of intra-/inter- neural network FCs and activities from those of resting, low arousal state in the SN, DMN and CEN, during which intra- and inter-network FCs and activities of all three networks are low. SS activation is positively linked to the activity of the SN and negatively to that of the DMN, while, in parallel, it is associated with an initial decrease and a subsequent increase of the intra- network FC and activity of the CEN. The FC between the DMN and the CEN increases, while those between the SN and the CEN decrease, allowing time for frontal lobe strategy input and “proper” CEN activity and task decision. SN activation is linked to sensory hypersensitivity, “impaired” memory, and a switch from serial to parallel processing, while trait mindfulness is associated with FC changes promoting CEN activity and producing a “task-ready state”.

**Conclusion:**

SS activation is tightly connected to that of the SN, with stress hormones likely potentiating the intra-network FC of the latter, attenuating that of the DMN, and causing a biphasic suppression-to-activation response of the CEN, all adaptive changes favoring proper decisions and survival.

## 1 INTRODUCTION

The stress system consists of the Hypothalamic-pituitary-adrenal (HPA) axis and the brainstem Locus Caeruleus/Norepinephrine-Autonomic Nervous System (LC/NE-ANS) and the peripheral limbs of the latter, the sympathetic nervous system (SMN) and parasympathetic nervous systems (PMN) [1]. The stress system is activated when a stressor exceeds a certain threshold and through its chemical mediators, assists in the maintenance of behavioral and physical homeostasis of the organism. The accepted mechanistic model of the stress response includes the central nervous system (CNS) and peripheral effectors with the secretion of the respective neurohormonal mediators, mainly corticotropin-releasing hormone (CRH), arginine-vasopressin (AVP) and norepinephrine in the brain, and the hormones glucocorticoids, norepinephrine, epinephrine, CRH, and Interleukin-6 IL-6) in the periphery [1]. Central and peripheral effectors of the stress system influence the emotional and cognitive responses of the brain and the cardiovascular, respiratory, metabolic, and immune responses of the entire body to stress.

Using engineering-derived metaphors, the accepted model of the stress response in the CNS uses the idea that the brain can be modelled as a collection of multiple neural networks reminiscent of multiple interconnected organs [2]. According to this idea, the CNS integrates information received from the external and internal environment [3]. Stimuli that are received by sensory structures are accepted, stored, and processed in the CNS, to generate appropriate, integrated responses in accordance with many other inputs [3]. The comprehensive “wiring diagram” of these networks and their intra- or inter-neural connections form the so-called connectome [4]. The histological mapping of neural connectivity and connectional patterns within and between anatomically distinct brain regions of the mammalian brain is divided into three-dimensional scales: a macroscale (coarse inter-regional connectivity), a mesoscale (focus on neurons and projections) and a microscale (reconstructing full details of all synaptic contacts) [5].

In the CNS, the stress response includes the facilitation of neural pathways that subserve acute, time-limited adaptive functions, such as those pertaining to the fight, flight or freeze reaction such as arousal, alertness, vigilance, and focused attention, cognition, and inhibition of neural pathways that subserve acutely nonadaptive functions, such as reproduction, feeding and growth [6]. Successful adaptive responses can be specific to a stressor or can be relatively non-specific when a stressor of any kind exceeds a threshold magnitude [7]. The sustained inability of the stress system to properly regulate the adaptive response may impair growth, development, behavior, metabolism, cardiorespiratory function and the inflammatory and immune reaction, and potentially leads to various acute and chronic disorders [8].

Apart from constantly improving histological techniques, great advances in our understanding of the brain have taken place by the use of magnetic resonance imaging (MRI) techniques, specifically diffusion MRI (dMRI) and functional MRI (fMRI) [9]. Diffusion MRI (dMRI) utilizes the anisotropic water diffusion in brain white matter to produce micro-architectural detail of white matter tracts, as well as to provide information about white matter integrity [10]. fMRI techniques, on the other hand, allow detection of increased neural activity, either as increased local cerebral blood flow (CBF) or as changes in oxygenation concentration (Blood Oxygen Level Dependent, or bold contrast) [11]. Thus, through dMRI, we define structural connectivity between brain regions as the presence of white matter tracts physically interconnecting these regions. This is the key method for mapping the connectional structure of the brain at the macroscale [12]. On the other hand, resting state fMRI (rsfMRI) is the key method for examining the functional brain connectome in the absence of performing an explicit task [9, 13, 14]. In recent years, studies of the human brain using rsfMRI have revealed collections of distributed regions that exhibit low-frequency, temporally correlated bold signal fluctuations [15]. Thus, rsfMRI has become an important tool in the study of functional interactions within the human brain in the absence of overt behavior [16]. Arterial spin labeling (ASL) is a noninvasive fMRI technique that uses arterial water as an endogenous tracer to measure CBF, which provides reliable absolute quantification of CBF with higher spatial and temporal resolution than bold techniques [17].

Functional connectivity (FC) analysis is a statistical measure of correlation or covariation that describes the interactions between fMRI signals obtained from discrete (two or more) brain regions without any assumption about the direction of these correlations [12]. In addition, recent studies have highlighted the potential of exploiting the dynamic properties of FC analysis in studying brain functioning at different timescales [18]. Assessment of temporal correlations in fluctuations of bold signals across spatially separate but functionally related brain regions has given rise to studies on resting state FC from fMRI bold signal time series [19]. These have been obtained during resting-state/ task-free periods (resting state fMRI - rsfMRI) or while cognitive actions take place in the brain based on an induced stimulus by a certain task (task-based fMRI - tbfMRI) [19]. In practice, rsfMRI is used to explore the intrinsic functional segregation or specialization of brain regions/networks, while the tbfMRI is employed to identify brain regions that are functionally involved in the performance of a specific task [19, 20]. These studies have produced a great deal of new information about the macro-scale spatiotemporal organization of the brain [21].

In the cerebral cortex, the resting activity levels of neuronal populations are continuously fluctuating. When neuronal activity levels are measured by fMRI over a period of time across regionally separate populations, these populations are said to be functionally connected when temporal coherence in their activities is observed [22]. Based on the correlations between intrinsic low-frequency oscillations mediated by the underlying structural connectivity, functional connectivity analysis has demonstrated that widespread brain regions show temporal coactivation patterns, allowing the identification of networks with distinct functions [23]. A number of intrinsic connectivity networks (also known as “large-scale brain networks”) are now widely recognized, including the salience network (SN), a cingulate-frontal operculum system anchored in the dorsal anterior cingulate cortex (ACC) and frontoinsular cortex (FIC), a central executive network (CEN) which is a frontoparietal system anchored in the dorsolateral prefrontal cortex (PFC) and the lateral posterior parietal cortex (PPC), and the default mode and memory network (DMN) anchored in the posterior cingulate cortex (PCC) and medial PFC, with prominent nodes in the medial temporal lobe (MTL) and the angular gyrus [24-27]. Of the many networks identified in the human brain, three SN, CEN and DMN have turned out to be particularly important for understanding higher cognitive function, dysfunction and psychopathology, hence the use of the term “core neurocognitive networks” [27].

The SN is involved in detecting bottom-up salient events, reallocates attention resources and is relevant to attending to survival-relevant events in the environment, integrating and filtering relevant interoceptive, autonomic and emotional information. The CEN is mainly involved in external executive function, is engaged by many higher level cognitive tasks and is thought to be involved in adaptive cognitive functions. Finally, the DMN is related to internally directed self-referential processing and involved in attention to internal emotional states; it is typically deactivated during most stimulus-driven cognitive tasks [26, 27]. The so-called “triple network model of major psychopathology” posits that aberrant intrinsic organization (intra-connectivity) and inter-connectivity among the DMN, the SN, and CEN is characteristic of many psychiatric and neurological disorders, while it proposes that weak salience detection and mapping of goal-relevant external stimuli and internal mental events from, and into, the SN plays a major role in human psychopathology [27, 28].

Dynamic FC analysis of neuroimaging data can be further employed in studying how inter-regional connectivity strength and network configurations evolve over time, by searching within a connectivity time series to identify recurring patterns known as “dynamic connectivity states” that potentially contain information that is of behavioral significance, particularly in states related to other cognitive domains [29]. Application of fMRI in the study of stress as a model of a large-scale/diffuse network has the potential to provide valuable insights into how stress affects neural circuitry and information-processing, how structural and functional connectivity interrelate and evolve, and how they break down in stress-related diseases, are areas of particular interest [9]. Furthermore, anatomical hubs in a large-scale brain organization and communication and the properties of task related networks underlie emotion regulation and memory consolidation in various clinical conditions, such as when the acute phase of the response to stress has waned and the application of effective interventions for treatment of both psychologic and physical symptoms associated with stress, such as mindfulness (defined as “the learned ability to be open, accepting, and present in the moment”) [30, 31]. Employing FC analysis to examine longitudinal changes within individuals over time may be particularly informative in the context of long-term interventions, given that alterations of cognition and other brain functions have underlying neural mechanisms that remain unexplored [32]. Initiatives such as “Brain Research through Advancing Innovative Neurotechnologies” (“BRAIN”) launched by the National Institutes of Health (NIH) in 2014 aim to quantify complex human behaviors and integrate them with the brain neural maps and recordings of brain activity [33].

This review aims to provide a comprehensive synthesis of previously published information on the conceptual evolution, technological advances, and current understanding of stress at the level of the central nervous system, as well as to selectively discuss some recent updates in this field of research.

## 2 MATERIALS AND METHODS

### 2.1 Eligibility Criteria

Study eligibility criteria were predetermined by the authors. To prevent language bias, our search was not limited by language. Also, there was no date or country restriction. We included randomized controlled trials employing fMRI techniques in the study of psychological or physiological stress. Reviews, letters, abstracts, case reports, expert opinions, *in vitro* and animal experiments, or conference abstracts were excluded during our data selection. The review was not registered.

### 2.2 Information Sources

Medline, Scopus, Clinical Trials.gov, and Google Scholar search engines were used in the primary search prior to September 2022.

### 2.3 Search Strategy

The Medical Subject Headings (MeSH) database was used for identification of synonyms. The identified groups were combined by the Boolean “AND” and the terms utilized within these search categories were combined by the Boolean “OR”. The full search strategy used for Pubmed was: “stress, psychological” (MeSH Terms) OR “stress, physiological” (MeSH Terms)) AND “Magnetic Resonance Imaging” (MeSH Terms) AND (clinical study (Filter) OR clinical trial (Filter) OR meta-analysis (Filter) OR observational study (Filter) OR randomized controlled trial (Filter). This was adapted appropriately for the rest of the databases.

### 2.4 Selection Process

Reference lists of selected articles were used to find additional studies that were not retrieved in the initial search. Conference abstracts were not searched on the principle that they do not contain enough data for quality assessment.

### 2.5 Data Collection Process

Two independent reviewers (GP) and (GPC) used the Medline, Scopus, Clinical Trials.gov, and Google Scholar search engines in primary search prior to September 2022.

### 2.6 Data Items-synthesis Methods

Subjects, interventions, fMRI techniques, and results were sought from each study included in the present systematic review, as demonstrated in Table **1**.

### 2.7 Study Risk of Bias Assessment

Risk of bias assessment was performed independently by the two researchers by employing the Jadad 5 point scale applied to each individual study [34]. Quality assessments were conducted separately by the authors, while any discrepancies were discussed and agreed on. All studies were of good quality.

## 3 RESULTS AND DISCUSSION

### 3.1 Study Selection

After the initial search and exclusion of duplicates, all documents obtained were screened independently by the two reviewers (GP and GPC). In brief, following the terms listed above, the electronic databases search resulted in 1539 articles, which were then checked for duplicates. After duplicates were detected and excluded, the list was restricted to 1240 articles that were screened by the two reviewers regarding their eligibility based on the relevance of their title and abstract to the subject (screening check). Following screening, 202 studies were further examined based on their title and abstract according to the eligibility criteria (first eligibility check). Following this examination, 53 full-text articles remained for further assessment of their full text based on the defined inclusion and exclusion criteria (second eligibility check). Among these articles, 25 were excluded for the following reasons: study type did not meet the eligibility criteria and/or the purpose of this systematic review. Finally, 28 out of 53 full-text articles were considered eligible and included for qualitative synthesis [35-62]. No additional records were derived through other sources (manual research, reference lists of other papers). During the entire procedure, each reviewer remained blinded to the other investigator’s selection. The study selection process was documented in the respective flow chart shown in Fig. (**1**).

The characteristics and the main outcomes of the selected studies are summarized in Table **1**. Due to the large heterogeneity of the studies, no formal meta-analysis or sub-analysis could be performed, however, a qualitative integration of currently available knowledge was attempted.

We found that, generally, in the resting, low arousal state, the stress system and the 3 core neural networks are relaxed and their functional intra- and inter-connectivities are low. In contrast, during the acute, high arousal state, the stress system and the networks are activated and/or suppressed with, respectively, high or low intra- functional connectivity, while their functional inter-connectivities are high.

## 4 DISCUSSION

To our knowledge, this is the first systematic review of the literature describing the stress response of the brain by employing fMRI techniques following acute stress interventions. Two of the assessed studies have shown that there is activation of specific limbic and midbrain regions, such as the left striatum and thalamus, the caudate, the right ventral prefrontal cortex, the left insula and putamen, the left hipppocampal and para-hippocampal regions, and the posterior cingulate cortex [35, 36]. In another study, there were stress-induced increases in FC between the amygdala and the medial prefrontal cortex, the posterior cingulate cortex, the putamen, caudate and thalamus [44]. Furthermore, the right ventral prefrontal cortex along with the right insula, putamen and anterior cingulate region showed sustained activation after task completion in subjects self-reporting higher stress levels [36]. Additional FC data showed that acute stress results in increased activity in the *cornu ammonis* and dentate gyrus of the hippocampus, which also predicted subsequent increases in the activity of the left insula and left midbrain, while the increases of the left thalamus activity predicted subsequent increases in the activation of all three hippocampal subregions (*cornu ammonis*, dentate gyrus and subicular complex); interestingly increased activity of the right thalamus predicted higher degrees of activity in the dentate gyrus after stress exposure [55]. These brain structures and their interrelations are involved in emotional information processing and storage as well as habitual responses, while the authors speculated that the altered effective connectivity in the thalamus-hippocampus-insula/midbrain circuit might be related to the encoding of salient negative information after acute social stress in the healthy individuals to better cope with similar future stressful events [55].

Early and fast stress responses *via* catecholaminergic pathways may have spatially selective effects on a network of brain regions that comprise the SN, which is often conceptualized as including the amygdala, the anterior middle cingulate cortex, the dorsal anterior cingulate cortex, the anterior insula, the thalamus, the temporo-parietal cortices, the striatum and the brainstem [52]. Particularly, the insula is a multifunctional, integrational brain region that, among other functions, serves in the control of the SN [63]. Of note, acute stress can lead to increased activity in a posterior visual cortex network, that, along with the differences in limbic and paralimbic regions (including the insula, dorsal-anterior and middle cingulate cortex, and striatum), comprise the processing areas of SN and are indicative of a state of sensory hypersensitivity under stress [39].

Functional connectivity analysis has also been employed to assess the adaptive recovery from an acute stress event and has provided data showing that following acute stress there is increased amygdala functional connectivity with three cortical midline structures - core constituents of the ‘default mode network (DMN), the posterior cingulate cortex and precuneus (areas implicated in autobiographical memory processes), and the medial prefrontal cortex, while an hour after application of an acute stressor, changes in the amygdala FC were detected with cortical midline structures involved in the processing and regulation of emotions, as well as autobiographical memory [37]. Of note, while the DMN is typically deactivated during most stimulus-driven cognitive tasks -as is the case during acute stress-, it is also hypothesized to provide the infrastructure for integrating past, present and future events related to the self [64]. Increased amygdala connectivity with these DMN regions could reflect stress-induced facilitation of self-evaluative processes under or after emotionally salient experiences, that would enable us to reflect on and learn from past experiences, which is essential for adaptively coping with future challenges [37]. When assessing trait mindfulness, *i.e*., individual’s natural or innate mindfulness tendency) as a paradigm of a measure of better coping with stress, the FC of DMN was negatively correlated with the FC of the other networks, while there was significant decoupling between the anterior and posterior components of the DMN with relation to mindful attention scale [65, 66]. Furthermore, it was shown that individuals with high mindfulness trait showcase more time in high within-network FC and lower FC between task-positive networks (SN and CEN) and the DMN [67]. Furthermore, another group showed that inter-individual differences in the degree of change in dynamic FC states after stress induction were related to the cortisol response and trait mindfulness, suggesting a potential novel pathway *via* which mindfulness might moderate the stress response [61].

Studying the combination of stress and a parallel task by comparing with a control group, the stress group showed a greater increase in neural activation in the anterior prefrontal cortex, a brain area associated with parallel processing, when performing two tasks simultaneously than when performing each task alone [41]. The findings are in line with the idea that stress seems to trigger a switch from serial to parallel processing in demanding dual-tasking situations. In addition, stress increases the choice pattern towards immediately rewarding taste attributes, while reduced self-control was accompanied by increased FC between the ventromedial PFC and amygdala, and with the striatal regions that encode tastiness [48]. Stress-related changes lead to increased oxygenation and nutrition of the brain, heart, and skeletal muscles *via* an increase of cardiovascular (CV) tone and respiratory rate, and catabolism, which in turn feedback and increase the inhibition of reproduction, feeding and growth, as they facilitate the “fight or flight” reaction [1, 68]. Furthermore, stress was associated with reduced connectivity between the ventromedial and dorsolateral PFC regions linked to self-control success [48]. Regarding learning, data suggest that individuals with strong cortisol responses to stress display less medial prefrontal cortex activity on fMRI during encoding of schema-related information and that their performance deteriorates if they rely on the hippocampus instead, while stress reduces the separation between brain connectivity patterns for learning-related and novel information, respectively, and is associated with impaired memory performance [54]. This should be considered along with fMRI data that demonstrate that acute stress selectively prioritizes the storage of threat stimuli that are presented outside the focus of attention [53]. The relevant fMRI neuroimaging results suggested that this threat-related memory enhancement after stress might be caused by threat-specific decoupling between amygdala and dorsal lateral prefrontal cortex during encoding [53]. Furthermore, when Kohn *et al* investigated how speed and performance in a cognitive task may be modulated under acute stress and how this is influenced by stress-related large-scale network dynamics, increased accuracy in stress was significantly associated to increasing activity in a set of brain regions that encompassed bilaterally the inferior frontal gyrus and the dorsolateral prefrontal cortex, the pregenual anterior cingulate cortex, the pre-supplementary motor area, extending into the anterior midcingulate cortex, bilateral angular gyrus and precuneus, which are parts of the ‘central executive network’ (CEN) [52]. The study results indicate that acute stress changes the state of brain connectivity in the SN and the CEN, increasing and decreasing the functional connectivity respectively, with the CEN suppressed in acute stress and upregulated in the aftermath of stress [52].

Interestingly, individuals showing an upregulation of the SN and down-regulation of the CEN under stress, displayed increased speed, but decreased performance quality, while individuals who upregulated CEN under stress showed improved performance [52]. This could potentially explain why certain executive control tasks may show an improvement under stress and what role inter-individual differences in large-scale network balance may play in the modulation of the performance of such tasks [52]. Of note, a novel fMRI neuroimaging approach, functional proton magnetic resonance spectroscopy (fMRS) that facilitates *in vivo* measurement of neurochemistry at time scales less than one minute, showed that acute stress attenuated working memory-induced glutamate modulation in the left dlPFC, a part of CEN, when compared to placebo [58].

The study of the endocrine part of stress system response has also been facilitated by fMRI studies. Recently, the novel Eigenvector centrality mapping was used to identify brain hubs involved in acute stress processing and showed an immediate, stress-driven change in whole-brain network topology that was increased in a cluster peaking in the thalamus, which was connected to regions across the entire brain, that was more pronounced in participants who also showed stronger stress-related changes in subjective and to a lesser extent autonomic, and hormonal measures [59]. It has previously been shown that following acute stress, there is an increased mean arterial pressure that correlates with greater fMRI response amplitudes in the perigenual and mid-anterior cingulate cortex, medial and lateral regions of the pre-frontal cortex, the insula, the periaqueductal gray matter, and the cerebellum, areas associated with cardiovascular reactions to behavioral stressors and also in sensory, motor, and multimodal association areas of the cortex, in two areas of the basal ganglia (caudate and lenticular nucleus), and in the anterior and posterior thalamus [69]. It has also been posited that hypertensive or hypotensive attacks could be caused by excessive sympathetic or parasympathetic system outflow induced *via* CRH bursts in the central amygdala that activate a stress related fear response [6]. Of note, the hypothalamus is responsible for the production of hormonal effectors of the stress system that include the HPA axis hormones CRH, AVP, and the pro-opiomelanocortin-derived peptides alpha melanocyte-stimulating hormone (α-MSH) and ß-endorphin [70].

When assessing HPA axis function, it has been shown that high stress-induced cortisol responses are associated with increased stress sensitivity as assessed by a greater stress-induced increase in medial temporal activity and greater differential amygdala responses [49]. In contrast, high basal cortisol levels are related to relative stress resilience, as reflected by a lower stress-induced increase in amygdala activity and enhanced differential processing under stress [49]. Furthermore, it has been shown that following acute stress, there is a negative correlation between the amygdala and hippocampal/para-hippocampal brain activities and cortisol secretion [71]. This can be further supported by data that show that acute stress decreases the activation of the nucleus accumbens, a central part of the brain’s reward system, during subconscious processing of sexual stimuli, while increased cortisol secretion following stress was related to a greater nucleus accumbens activation [42]. Furthermore, it has been shown that following administration of 10 mg of hydrocortisone in men, there is reduced “positive” functional coupling of the amygdala to brain regions involved in the initiation and maintenance of the stress response, *i.e*., the locus caeruleus, hypothalamus, and hippocampus, while significantly reduced the correlated activity observed between the amygdala and the middle frontal and temporal gyri; brain regions known to be involved in executive control [38]. The hypothalamus, a major effector site of the stress system, is composed of several small essential nuclei, including the arcuate (ARC), paraventricular (PVN), supraoptic (SON), suprachiasmatic (SCN), dorsomedial (DMH), and ventromedial (VMH) nuclei, and the lateral hypothalamic area [72]. The PVN, which is located in the ventral diencephalon adjacent to the third ventricle, is a highly conserved brain region that is composed of heterogeneous parvocellular neurons, magnocellular neurons, and long-projecting neurons [72].

The locus caeruleus in the brainstem is the second major effector site of the stress response in the CNS and part of the arousal and autonomic nervous system secreting primarily norepinephrine [73]. Of note, control analysis did not show significant corticosteroid modulation of visual cortex coupling covering the occipital lobe, cuneus, calcarine, lingual gyrus, and fusiform gyrus, and the negative coupling of the primary visual cortex between the cerebellum and a region within the brain stem [38]. Thus, corticosteroids seem to “decouple,” or disconnect, the amygdala from the rest of the brain by reducing functional connectivity to regions that are positively, as well as negatively, correlated with its activity [38]. In addition, when assessing *via* fMRI the effect of mineralocorticoid receptor blockade on acute stress, stress after placebo promoted striatum-based learning at the expense of hippocampus-based learning, while FC analyses showed that this shift was associated with reduced amygdala-hippocampus and increased amygdala-putamen coupling [40]. In contrast, stress after mineralocorticoid receptor blockade prevented the shift toward striatal procedural learning, with significantly impaired performance [40]. The same group showed that effective stress induction and successful conditioning were accompanied by a stress-induced reduction of learning-related hippocampal activity for trace conditioning, while this effect in neural processing was absent in mineralocorticoid receptor-blocked subjects [45]. Furthermore, again by the same group, data have shown that stress-induced increases in cortisol lead to enhanced stimulus-response learning, accompanied by increased amygdala activity and connectivity to the striatum [50]. Importantly, this shift was prevented by an acute administration of the MR-antagonist spironolactone, suggesting the presence of a mechanism that adaptively allocates neural resources [50].

When assessing the effect of moderate to severe emotional neglect and abuse in childhood, a significant deactivation during stress in limbic regions, such as the hippocampus, anterior cingulate cortex, amygdala and parahippocampal gyrus and also a correlation between hippocampus deactivation with the salivary cortisol response was found, while oxytocin administration produced a stress-buffering effect in control subjects [43]. Interestingly, CRH+ neurons also release AVP and oxytocin, both of which can sufficiently activate ACTH secretion even without the presence of CRH [74]. In contrast to their roles in the anterior pituitary, AVP and oxytocin inhibit the HPA axis through dendritic release within the PVN [74]. Subsequent studies showed that history of emotional abuse was positively associated with stress-induced changes in connectivity between the amygdala and hippocampus, an effect that was moderated bythe administration of intranasal oxytocin [44]. Summarizing the potential effect of oxytocin in modulating chemosensory-induced stress responses, a recent RCT showed that oxytocin selectively diminished chemosensory-induced behavioral biases in fear perception and neural responses to stress-associated odors in the amygdala, hippocampus, and anterior cingulate cortex [56]. In addition, FC analyses delineated a reinstated functional coupling between the stress-sensitive anterior cingulate cortex and the fusiform face area, after oxytocin treatment, highlighting an intensified interaction between olfactory and visual networks [56].

In addition, when assessing acute stress induction *via* NMDA receptor blockade by ketamine, fMRI data showed that, indeed, ketamine mimics a stress-like response with a characteristic spatiotemporal profile of a transient prefrontal hyperperfusion and a dose-related reduction of relative hippocampal perfusion [46]. This pharmacologic stress response in FC analysis showed hyperconnectivity between the hippocampus and the occipital, cingulate, precuneal, cerebellar and basal ganglia regions, suggesting a topographically dissociable change in corticohippocampal FC [46]. In contrast, when evaluating the beneficial effect to stress of pharmacologic agent Nx4, as opposed to placebo, differential activation was found in the cortico-medial portion of the amygdala, an area known to directly influence the autonomic nervous system and modulate the release of stress hormones *via* afferents to the hypothalamus, thus supporting the hypothesis that cortico-medial amygdala influences behavioral responses to stress and causes a perceived feeling of stress [60]. As it has been previously shown, the amygdala is activated following negative stimuli and directly influences the autonomic nervous system, modulating the release of stress hormones *via* afferents to the hypothalamus [75].

By employing fMRI and FC analysis, higher neuroticism trait levels predicted a greater response of the amygdala to an appropriately stressful stimulus, but this effect depended on the current stress state of the individual [47]. When assessing the levels of burnout following stress rehabilitation, the striatal activity decreased as a function of improved levels of burnout, while no significant association between burnout level and working memory performance was found; the authors posited that frontostriatal neural responses related to working memory were modulated by burnout severity [51]. Furthermore, by employing fMRI it was shown that ventral striatum and orbitofrontal cortex (OFC) responses to positive task feedback of the reward circuitry in the aftermath of stress are increased in healthy male controls, as compared to at-risk for schizophrenia individuals [57]. It was posited that chronic stress may be associated with physical, behavioral and/or neuropsychiatric manifestations, with the respective pathogenesis attributed to increased secretion and effects of the major stress mediators in the context of a vulnerable background [6].

**Fig. (2):**
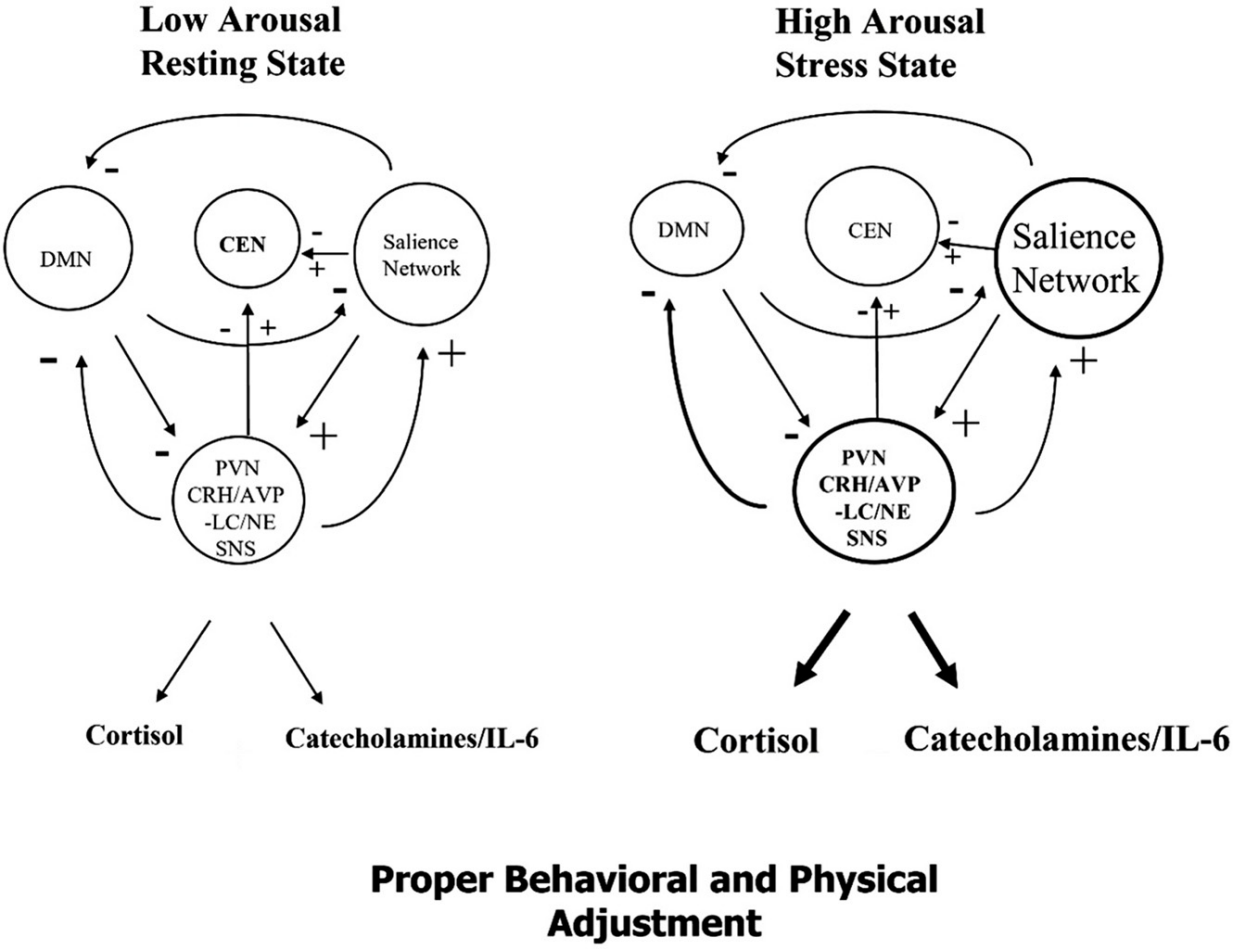
A heuristic presentation of the interplay between the stress system (PVN-CRH and LC/NE-SNS) and the salience (SN), default mode (DMN), and central executive (CEN) networks. The interactions of the stress system and the above 3 large scale neuronal networks in the human brain are shown in the low arousal resting state (**left**) and in the acute, high arousal stress state (**right**). Generally, in the resting, low arousal state, the stress system and the core neural networks are relaxed and their functional intra- and inter-connectivities are low. In contrast, during the acute, high arousal state, the stress system and the networks are activated and/or suppressed with respectively high or low intra- functional connectivity, while their functional inter-connectivities are high. **Left:** In the resting state, the stress system and the individual inherent intra- and inter- functional connectivities between the DMN, SN, and CEN, as well as their activities, are low, with the brain being metaphorically, in “a relaxed” state. **Right:** During the acute, high arousal stress state, the stress system and the SN are activated in a reciprocal fashion (stimulating each other’s activities). The intra-network functional connectivity and activity of the SN are increased, while its inter-network functional connectivity with the CEN and the DMN is also increased, leading to an initial suppression of the activities of both of these networks. In a subsequent second time phase, the stress system and the SN stimulate the intra-network FC and activity of the CEN, presumably allowing time for frontal lobe strategy input and a proper decision for action to be made. Ultimately, the DMN and the CEN activities are anti-correlated during acute stress system and SN activation. During mindfulness, or in subjects with “a high mindfulness trait”, the intra-network connectivity of the DMN is increased and somehow restrains both the stress system and SN responses to an acute stressor(s), limiting the initial negative effect of the stress system and the SN on the activity of the CEN. This usually propitious interrelation of the stress system and the 3 large scale neuronal networks allows better performance of tasks, and, hence, represents a good “task-ready” state. + = Stimulation of activity, - =Inhibition, +/- = either stimulation or inhibition.

In summary, recent stress studies have shown that acute stress induces re-allocation of neural resources entailing increases in intra-SN connectivity at the cost of decreases in DMN and, initially, CEN inter-connectivities, with SN signaling a switch between internal (DMN) and external (CEN) modes of thought (Fig. **2**) [61, 62, 67, 76]. Upregulation of the SN at an initial cost of the CEN may explain the potentially detrimental effect of stress on executive control tasks in certain maladaptive stress situations [77]. Ultimately, during stress, the FC between the DMN and the CEN increases, while those from the SN to the DMN and the CEN decrease, allowing, thus, ample time for frontal lobe input and proper task decision making, as shown in a group of adolescents [62]. Recently, a mindfulness trait was associated with relatively increased intra-network FC in the DMN and a decreased inter-network FC between the SN and the DMN, a decreased response of the HPA axis, and a better “task ready state” in the CEN [61, 67]. This probably helps explain the protective effects of mindfulness in coping with stressors [78]. The main limitation of this study is the heterogeneity of the studies included in our analysis. These were: the varying sample sizes, the differences in the age range of the subjects, the scanner types employed, and the experimental procedures used in the induction of acute stress. This heterogeneity allowed only a qualitative, albeit useful, synthesis of the information.

## CONCLUSION

In conclusion, fMRI techniques provide *in vivo* data on the CNS changes that take place during acute stress, particularly in the interconnected SN, CEN and DMN large-scale brain networks. Exploring how low arousal resting-state functional organization of the CNS is affected by acute stress and how FC changes are valid functional dynamic neuroimaging research questions. Studies so far show that stress causes an activation of the SN and a state of sensory hypersensitivity and parallel tasking. The organization and the FC of the separate core neural networks involved in the stress response can be further evaluated using different fMRI techniques that also allow interpretations of brain function at different timescales. Of particular interest are studies assessing the progressive development of an expanded brain “stress network” as part of normal child and adolescent development. We hope that it will provide invaluable information on the key psychobiological mechanics of human ontogeny.

## Figures and Tables

**Fig. (1):**
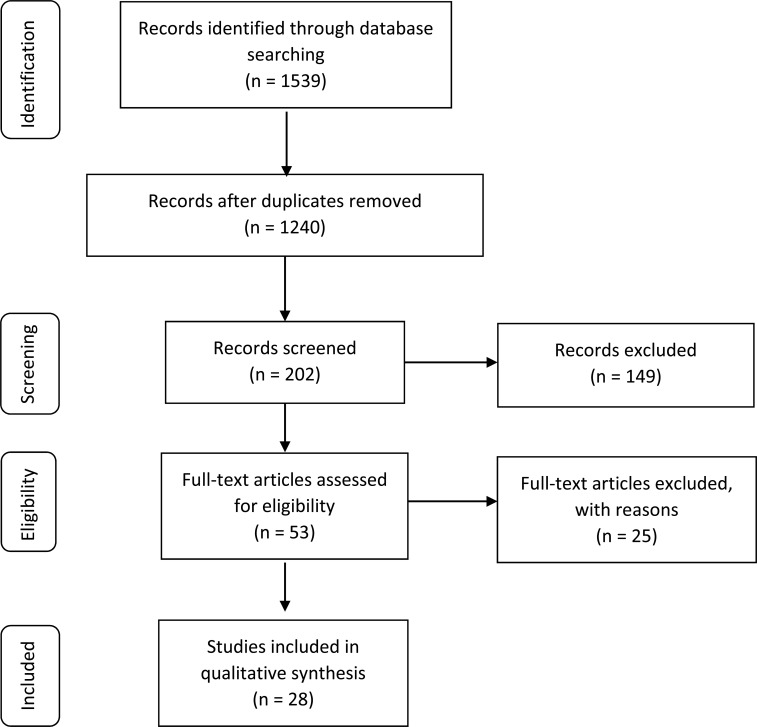
Study selection flow-chart.

**Table 1:** Summaries of the studies derived from the systematic search.

**S. ** **No.**	**Study**	**Subjects**	**Intervention**	**fMRI Technique**	**Results**
1	Sinha *et al*. (2004) [35]	• 8 adults (32.6 ± 5.7yrs)	Acute stress (self-reported stress stimuli evaluated on a Likert scale)	Resting-state fMRI (1.5T)	• Increased activation in specific limbic and midbrain regions (left striatum and thalamic regions, bilateral caudate and putamen, left hipppocampal and para-hippocampal regions, and the posterior cingulate).
2	Wang *et al*. (2005) [36]	• 25 stress group (24.1 ± 2.8 yrs) • 7 control (23.4 ± 1.3yrs)	Acute stress (mental arithmetic task)	Resting-state Arterial spin-labeling perfusion (ASL) fMRI (3.0T)	• Increased activation in the ventral right prefrontal cortex and left insula and putamen areas.
3	Veer *et al*. (2011) [37]	• 18 stress group (23.94 ± 3.12 yrs) • 20 control (23.95 ± 2.52 yrs)	Acute stress (Trier Social Stress Test)	Resting-state fMRI (3.0T)	• Increased amygdala functional connectivity with three cortical midline structures – core constituents of the default mode network: the posterior cingulate cortex and precuneus areas, and the medial prefrontal cortex. • One hour after the acute stress, changes in amygdala functional connectivity were detected with cortical midline structures involved in the processing and regulation of emotions, as well as autobiographical memory.
4	Henckens *et al.* (2012) [38]	• 20 hydrocortisone • 23 control (All male 19-28 yrs, median 21)	Pharmacologic stress (10 mg hydrocortisone)	Resting-state fMRI (1.5T)	• Reduced “positive” functional coupling of the amygdala to brain regions involved in the initiation and maintenance of the stress response.
5	Qin *et al.* (2012) [39]	• 22 stress group (21.65 ± 3.73 yrs) • 22 control (22.71 ± 4.01 yrs)	Acute stress (custom protocol)	Resting-state fMRI (3.0T)	• Increased activity in a posterior visual cortices network. • Differences in limbic and paralimbic systems including insula, dorsal-anterior and middle cingulate cortex, and striatum (salience processing network).
6	Schwabe *et al.* (2013) [40]	• 38 stress group (19 placebo, 19 spironolactone) • 37 control (19 placebo, 18 spironolactone) (24.6 ± 0.3 yrs)	Acute Stress (probabilistic classification learning task) after placebo vs stress after mineralocorticoid receptor blockade	Resting-state fMRI (3.0T) whole-brain analyses and region of interest (ROI) analyses	• Stress after mineralocorticoid receptor blockade prevented the shift toward striatal procedural learning with significantly impaired performance.
7	Gathmann *et al.* (2014) [41]	• 19 stress group (23.69 ± 5.00 yrs) • 19 control (24.06 ± 5.07 yrs)	Acute stress (Trier Social Stress Test) and a parallel task	Task-based fMRI (7.0T)	• Stress elicited a greater increase in neural activation in the anterior prefrontal cortex when performing two tasks simultaneously than when performing each task alone.
8	Oei *et al.* (2014) [42]	• 20 stress group (22.42 ± 3.25 yrs) • 17 control (21.50 ± 2.90 yrs)	Acute stress (Trier Social Stress Test)	Resting-state fMRI (3.0T)	• Decreased activation of the nucleus accumbens, a central part of the brain’s reward system, during subconscious processing of sexual stimuli.
9	Grimm *et al.* (2014) [43]	• 14 stress group (29.5 ± 4.5 yrs) • 17 control (29.0 ± 5.1 yrs)	Acute stress (Montreal Imaging Stress Test) in subjects who experienced moderate to severe emotional neglect and abuse in their childhood	Resting-state fMRI (3.0T)	• Significant deactivation during stress in limbic regions such as the hippocampus, anterior cingulate cortex, amygdala and parahippocampal gyrus and also a correlation between hippocampus deactivation with the salivary cortisol response, without the stress-buffering effect following oxytocin found in control subjects.
10	Fan *et al.* (2015) [44]	• 31 male within-subject crossover design (28.2 ± 4.7 yrs)	Acute stress (Montreal Imaging Stress Test)	Task-based fMRI (3.0T)	• Stress-induced increases in functional connectivity between amygdala and medial prefrontal cortex, posterior cingulate cortex, putamen, caudate and thalamus. • Regression analysis showed that history of emotional abuse was positively associated with stress-induced changes in connectivity between amygdala and hippocampus an effect that was moderated after the administration of intranasal oxytocin.
11	Vogel *et al.* (2015) [45]	• 101? stress group (? placebo, ? spironolactone) • ? control (? placebo, ? spironolactone) (21.9 ± 2.9 yrs)	Acute stress (Socially Evaluated Cold Pressor Test MRI scanner–compatible version) and successful conditioning	Task-based fMRI (1.5T)	• Stress-induced reduction of learning-related hippocampal activity for trace conditioning. • Absent in mineralocorticoid receptor blocked subjects.
12	Khalili-Mahani *et al.* (2015) [46]	• 12 male single blinded, randomized, placebo-controlled crossover design (19-36 yrs)	Pharmacologic stress (NMDA receptor blockade by ketamine)	Resting-state fMRI Arterial spin-labeling perfusion (ASL) (3.0T)	• Mimics a stress-like response with a characteristic spatiotemporal profile. • Transient prefrontal hyperperfusion. • Dose-related reduction of relative hippocampal perfusion. • Emerging hyperconnectivity between the hippocampus and the occipital, cingulate, precuneal, cerebellar and basal ganglia regions.
13	Everaerd *et al.* (2015) [47]	• 118 male randomized, controlled cross-over design (22.0 ± 2.6 yrs)	Acute stress and neuroticism trait assessment	Task-based fMRI (1.5T)	• Higher neuroticism trait levels predicted a greater response of the amygdala to an appropriately stressful stimulus.
14	Maier *et al.* (2015) [48]	• 51 male • ? stress group • ? control (21 ± 2 yrs)	Acute stress Socially Evaluated Cold Pressor Test	Task-based fMRI (3.0T) and FC analysis	• Stress increased choice pattern towards immediately rewarding taste attributes. • Reduced self-control were accompanied by increased functional connectivity between ventromedial prefrontal cortex (vmPFC) and amygdala and striatal regions encoding tastiness.
15	Henckens *et al.* (2016) [49]	• 120 male randomized, controlled cross-over design (18-30 yrs)	Acute stress (custom)	Task-based fMRI (1.5T)	• High stress-induced cortisol responses were associated with a stronger stress-induced increase in medial temporal activity and greater differential amygdala responses (stress sensitivity). • High basal cortisol levels were associated with lower stress-induced increase in amygdala activity and enhanced differential processing under stress (stress resilience).
16	Vogel *et al.* (2017) [50]	• 101? stress group (? placebo, ? spironolactone) • ? control (? placebo, ? spironolactone) (21.9 ± 2.9 yrs)	Acute stress (Socially Evaluated Cold Pressor Test MRI scanner-compatible version)	Task-based fMRI (1.5T)	• Stress-induced increases in cortisol lead to enhanced stimulus-response learning, accompanied by increased amygdala activity and connectivity to the striatum. • This shift was prevented by an acute administration of the MR-antagonist spironolactone.
17	Gavelin *et al.* (2017) [51]	• 10 cognitive training group (41.50 ± 7.18) • 11 control (39.09 ± 9.44 yrs)	Diagnosis of stress-related exhaustion disorder - 24-week stress rehabilitation	Task-based fMRI (3.0T)	• Striatal activity decreased as a function of improved levels of burnout, while no significant association between burnout level and working memory performance was found.
18	Kohn *et al.* (2017) [52]	• 120 randomized, controlled cross-over design	Acute stress (custom)	Task-based fMRI (1.5T) and FC analysis	• Increased accuracy in stress was significantly associated to increasing activity in a set of brain regions that encompassed bilateral IFG and DLPFC, the pregenual ACC, pre-SMA, extending into the aMCC, bilateral angular gyrus and pre-cuneus.
19	Luo *et al.* (2018) [53]	• 27 stress group • 26 control (22.22 ± 2.89 yrs)	Acute stress (Socially Evaluated Cold Pressor Test MRI scanner-compatible version)	Task-based fMRI (1.5T)	• Acute stress selectively prioritizes the storage of threat stimuli that are presented outside the focus of attention.
20	Vogel *et al.* (2018) [54]	• 51mixed design with the between-subjects factor treatment (25.0 ± 0.48 yrs)	Acute stress (Trier Social Stress Test)	Task-based fMRI (3.0T)	• Strong cortisol responses to stress display less medial prefrontal cortex activity. • Stress reduces the separation between brain connectivity patterns for learning related and novel information, respectively, and is associated with impaired memory performance.
21	Chang *et al.* (2019) [55]	• 30 randomized, controlled cross-over design (20.6 ± 2.0 yrs)	Acute stress (Trier Social Stress Test)	Resting-state fMRI (3.0T) and effective connectivity analysis	• Acute stress resulted to increased activity in the cornu ammonis and dentate gyrus that predicted subsequent increases in activation of the left insula and left midbrain activity.
22	Maier *et al.* (2019) [56]	• 50 randomized, double-blind, placebo-controlled, crossover design (24.54 ± 3.09 years)	Acute stress (Trier Social Stress Test)	Task-based fMRI (3.0T) and FC analysis	• Oxytocin selectively diminishes chemosensory-induced behavioral biases in fear perception and neural responses to stress-associated odors in the amygdala, hippocampus, and anterior cingulate cortex. • FC analyses delineates a reinstated functional coupling between the stress-sensitive anterior cingulate cortex and the fusiform face area, after oxytocin treatment.
23	van Leeuwen *et al.* (2019) [57]	• 18 control-no-rsstress (35.4 ± 2.0 yrs) • 20 control-stress (33.1 ± 2.0 yrs) • 18 sibling-no-stress (33.4 ± 2.6) • 18 sibling-stress (32.6 ± 1.7)	Acute stress (Trier Social Stress Test)	Resting-state & Task-based fMRI (3.0T)	• Ventral striatum and orbitofrontal cortex (OFC) responses to positive task feedback of the reward circuitry in the aftermath of stress are increased in healthy male controls, as compared to in at-risk for schizophrenia individuals.
24	Woodcock *et al.* (2019) [58]	• 19 double-blind, within-subject crossover conditions (27.5 ± 3.9 yrs)	Pharmacologic stress (yohimbine and hydrocortisone)	Task-based fMRI (3.0T) and functional proton magnetic resonance spectroscopy (fMRS)	• Attenuated working memory-induced glutamate modulation in the left dorsolateral prefrontal cortex (dlPFC).
25	Reinelt *et al.* (2019) [59]	• 33 stress group (25.45 ± ? yrs) • 34 control (26.18 ± ? yrs)	Acute stress (Trier Social Stress Test)	Resting-state fMRI (3.0T) and Eigenvector centrality mapping	• Stress-driven change in whole-brain network topology. • Increased in a cluster peaking in the thalamus • Cluster connected to regions across the whole brain. • More pronounced in participants that showed stronger stress-related changes in subjective and to a lesser extent autonomic, and hormonal measures.
26	Hermann *et al.* (2020) [60]	• 20 Nx4 (43.2 ± 10.00 yrs) • Placebo (43.7 ± 9.61 yrs)	Acute stress (Harriri stress test) and pharmacologic agent Nx4	Resting-state & task-based fMRI (3.0T)	• Differential activation in the cortico-medial portion of the amygdala.
27	Teng *et al.* 2022 [61]	• 41 male participants of Asian ethnicity (18-35y)	Acute stress (Trier Social Stress Test)	Resting-state fMRI (3.0T)	• Acute stress led to significant decreases in the prevalence of FC state previously associated with mindfulness. • Higher trait mindfulness was associated with attenuated affective and neuroendocrine stress response, and smaller decreases in the mindfulness-related FC.
28	Corr *et al.* 2022 [62]	• 79 adolescents (12.8 ± 2.2 yrs)	Acute stress (Montreal Imaging Stress Task)	Resting-state fMRI (3.0T)	• FC was increased between DMN and CEN regions and decreased between the SN and the DMN and CEN.

## LIST OF ABBREVIATIONS

ASL: Arterial Spin Labeling
AVP: Arginine-vasopressin
CBF: Cerebral Blood Flow
CEN: Central Executive Network
CNS: Central Nervous System
CRH: Corticotropin-releasing Hormone
CV: Cardiovascular
FC: Functional Connectivity
FIC: Frontoinsular Cortex
HPA: Hypothalamic-pituitary-adrenal
MRI: Magnetic Resonance Imaging
PCC: Posterior Cingulate Cortex
PFC: Prefrontal Cortex
PPC: Posterior Parietal Cortex
PVN: Paraventricular
SCN: Suprachiasmatic
SMN: Sympathetic Nervous System
SN: Salience Network
SON: Supraoptic

## STANDARDS OF REPORTING

PRISMA guidelines and methodology were followed.

## SUPPLEMENTARY MATERIAL

PRISMA checklist is available as supplementary material on the publisher’s website along with the published article.
